# American Academy of Dental Science

**Published:** 1894-04

**Authors:** 

**Affiliations:** American Academy of Dental Science


### AMERICAN ACADEMY OF DENTAL SCIENCE.

The regular monthly meeting of the American Academy of Dental Science was held at the Boston Medical Library Association Rooms, Wednesday evening, December 6, 1893, at 7.30 o'clock, President Smith in the chair.

The subject of discussion for the evening was "Orthodontia."

*Dr. H. A. Baker.*—Mr. President and Gentlemen,—Dr. Ainsworth and I do not claim to bring anything new before this meeting. We merely show a method by which regulating is made easy. We believe this method to be adapted to a greater variety of cases than any other of which we are familiar. It has to do with bands, a spread-plate, and retaining spurs.

The band is, in many ways, original with Dr. Ainsworth. He has already brought it before this society. I have of late adopted it in most cases.

The spread-plate differs from others in this respect, that the screw is made of German silver and the nuts are vulcanized into the rubber. With this appliance, you can push the teeth on one side more than on the other by properly slanting the screw; this can be done although the plate is split equally on both sides. Dr. Ainsworth, I think, claims the originality of this arrangement.

The spur I believe to be original with myself, and is adapted to the retention of a great many cases, after having been regulated. In many instances no other retainer need be used. It consists of a little spur or pin projecting from a cavity in one tooth, and resting on the adjacent tooth, thus holding the teeth in position. It is particularly useful in holding a tooth after being rotated, or in holding one or more teeth in line. Many cases have been retained by these spurs alone, without other appliances. I have here the models of a few cases, which I will show.

Nearly all these cases have been regulated by means of Dr. Ainsworth's band. This case has a right central turned a quarter way round, and a right cuspid placed outside of the arch. The irregular teeth are now in good line, and held in position by spurs. Here is another case, which is very much out of line, both as regards the upper and under teeth; the upper ones only have been regulated, and are now held in place by retaining spurs. Here is another case, in which the right cuspid is very much out of line, and the arch contracted. In this case I used the band in connection with the spur as a retainer. This case shows a separation of the front teeth. The under teeth seemed to throw the upper ones out, making a space of about one-sixteenth of an inch between the incisors. This condition was corrected by the use of the band, which threw back the front teeth, both upper and lower. These teeth were retained in position by a wire hook passing around the upper centrals. I do not claim that they will remain in place. There is a cause for the separation of these teeth, and I do not know whether or not there is any remedy for it. I have here a bad case of protruding upper jaw, or a much receding lower one. This case is in my own family, and no one wishes it corrected more than I do. My plan of treatment is to put in place bands on both upper and lower teeth, and spread the arches by tying to the bands all the teeth in front of the molars. Then to take four square nuts, and solder a hook to each, and screw them on to the bands. Elastics are to be slipped over these hooks. The child is to wear the appliance constantly. You will see that the tendency of this arrangement will be to draw the upper jaw back and the lower one out, and so jump the bite. I don't know whether this apparatus will interfere with eating or not. I will try and explain on this model how teeth are retained by spurs. The right central and right lateral are both twisted. The right central was turned to place, and a spur put in the mesial surface of the right lateral, the spur holding the central in place. After twisting the right lateral to place, it was retained by drilling into the distal side of the right lateral, and putting in a spur which rested on the cuspid. Another spur in the mesial surface of the right central rested on the palatal surface of the left central.

*Dr. Andrews.*—Do I understand that these spurs are two little pieces of gold, connected with the band?

*Dr. Baker.*—No. The gold spurs project from the cavity in the tooth, about one-sixteenth of an inch, and rest on an adjacent tooth. They are made by cutting a strip from a piece of thick plate, and putting it in a port-polisher and working it down to the desired size. To insert the spur, I drill a cavity in the tooth, if there is not one there already, fill the cavity with cement, then insert the pin in cement. These pins will sometimes last a year or two, and there is no inconvenience from their use.

*President Smith.*—Do I understand that you have to drill a hole in a sound tooth in order to secure your spur?

*Dr. Baker.*—Usually a cavity already exists, but if there is none, I do not hesitate to drill one. These cavities can be readily filled again, in such a way that they will not decay. The only precaution to be used is to avoid drilling too near the pulp. I happened to discover these spurs in this way. I had a case some fourteen years ago which required extensive regulating. After the regulating was completed, the question of a retaining appliance presented itself. My patient told me that he would not wear anything which showed. I replied that I did not see how it was possible for me to put anything in which would satisfactorily retain the teeth, and at the same time be out of sight. My patient still held to his point, and as a result of careful study, I thought of these spurs. Although I put in his mouth four or five spurs, in only one case was I obliged to drill into a sound tooth.

*Dr. Andrews.*—How long have you used these spurs?

*Dr. Baker.*—Twelve or fifteen years. I brought the matter before the Massachusetts Dental Society several years ago. I do not claim that this method is original, but I do not know of its having been used. To illustrate the difference between this method and that of retaining plates, I will mention the following case: A patient of mine, whose teeth had been successfully regulated, wore a retaining plate for about six months. I found that the enamel was decidedly affected in the case of the lower teeth, and I came very near having a bad result. I removed the plate in time to prevent serious harm, and became convinced that it was better to have the teeth in their natural condition, with as little as possible in the mouth in contact with them. To my mind, the drilling of a cavity in a sound tooth is far less objectionable than a retaining plate, because the point of contact is much smaller.

*Dr. Ainsworth.*—The apparatus used in the correction of the cases which I present to-night is the same which I brought before the Academy about a year ago, and as to the details of this apparatus, I believe I may claim some originality. It was in use by me two or three years before I brought it before this society. The apparatus is a modification of the Patrick band, from which I failed to get much satisfaction. After discarding this, I tried a band having its ends soldered to the anchor bands which were about the molars. But the heating of the band necessary to this operation removed its spring temper, a most important quality. In seeking a way to overcome this difficulty, I devised this method. The ends of the band were provided with a thread and nuts, and were allowed to pass through tubes about a quarter of an inch in length, which were soldered to the sides of the anchor bands, these bands being placed upon the molars. Here is a case where the upper front teeth protrude very much, the arch being contracted at the bicuspids. The lower teeth strike one space too far back. My first step was to spread the upper arch so as to make a fair occlusion when the under teeth were closed one space further forward. After spreading the arch, I put the nuts at the rear ends of the tubes, and drew the front teeth in until the upper arch was in proper shape. I then instructed the patient to project the under jaw in closing the mouth, and relied upon her intelligence to accomplish the desired result. In this way the bite was successfully jumped. At first, especially at night, she found it difficult to keep her mouth closed in the new way. In the morning the occlusion would be more natural in the old way. She has entirely overcome that now. Had I used Dr. Baker's spurs on the laterals, they would have been held more positively, but the result is not unsatisfactory as it is. I use German silver for my band, but I know of no objection to using platinized gold. There is no one system of regulating which is invariably better than another. I believe the best system of regulating is that to which the operator is most accustomed. There is comparatively little trouble in moving teeth into proper position, but it is a great problem to hold them there. Here is a case of very prominent upper teeth, and it was very difficult to hold them in place after regulation, because the child did not appreciate what was being done for her.

*Dr. Mead.*—If a plate had been struck up and cemented over the tips of the front teeth, would not that have held them in position?

*Dr. Ainsworth.*—Such a plate would be effective, but also a great disfigurement.

*Dr. Mead.*—I have never had any trouble with this method. Once in a while the plate gets loose, and I replace it.

*Dr. Ainsworth.*—I think there is a great deal of value in the spur of which Dr. Baker speaks. In many cases a cavity already exists in which to insert the spur. I never hesitate, however, to drill one for this purpose. This seems to me a far less evil than to run the risk of damage from a less positive retaining appliance. Here is the material of which the band is made; it is all German silver. That for the anchor bands is wire rolled down very thin. Here are some seamless German silver tubes, made for me by the Spring Garden Metal Works of Philadelphia.

Here is a model showing a method which I pursue in the treatment of approximal decay in the molars of children. This case came to me with decay between the temporary molars. I cut a V-shaped opening between them, and thereby nearly obliterated the cavity. Seeing that trouble was coming between other temporary molars, I cut a V-shaped cavity between them all. I also caused the mesial surface of the sixth-year molar to be open to friction. This method has given me great satisfaction. In the chair, to-day, I examined the mouth of one of my own children, between whose temporary molars I had ground a V-space. I found that decay had been entirely stopped by the friction to which the teeth had been subjected.

Here is a case not in line with the subject of the evening, but of considerable interest to me. The upper front teeth were worn down nearly to the gum line. The pulps were alive, and so sensitive as to cause much trouble and inconvenience. I crowned all the teeth, using countersunk pin teeth set in firmly-fitting gold bands.

The work was finished a year and a half ago, and the teeth have done good service with nothing whatever to hold them but the band and the cement. One of them worked loose this summer, and I reset it; the others seemed as tight as though they were a part of the tooth itself. I have had occasion to set a number of such crowns upon teeth having live pulps, and always with a great deal of satisfaction.

*Dr. Wilson.*—In the case of Dr. Ainsworth's regulating appliance, I would like to ask, what is to prevent the anchor bands from getting up on to the gums.

*Dr. Ainsworth.*—That is a very important point. I wish to make it clear that the anchor bands are cemented on to the molars. They are of such thin metal that it is not necessary to file between the molars to get room to put them on. A wedge can be placed in both sides of the molar, and in two hours' time there will be room enough to pass this band through. Then I would use a very adhesive cement, and cement the band in place. They will occasionally come off, but not very often.

*Dr. Mead.*—Do you cement your bands and apply your wire at the same sitting?

*Dr. Ainsworth.*—Sometimes I would, and sometimes I would not. I consult my convenience and that of the patient. It is a good plan to cement the bands on one day, and put on the wire the next; in that way, you give the cement sufficient time to become thoroughly set. I do not take pains to make a perfectly fitting anchor band. I intend it shall be too large, so as to have a good body of cement to hold it.

*Dr. Mead.*—On leaving the house this afternoon, I took with me the models of what I call a "bull-dog mouth," and I brought also the appliance which I used in correcting it. I made a simple rubber plate to open the bite, so that the protruding under teeth could slide back within the uppers. By this plate also the lower teeth were brought in by repeated ligations. The upper laterals were brought out by the "McQuillen band," with elastics attached, and it was done in a few days. The whole thing was completed in three months. The models are here before you, along with the appliances used.

*Dr. Brackett.*—One of the difficult teeth to retain in a changed position is a superior lateral which has been prominent, and whose mesial side has been rotated outward. If I rightly understood Dr. Ainsworth, he made the statement that the spur was especially applicable to the retention of these teeth, and that it might always be inserted on the palatal side.

*Dr. Ainsworth.*—That is one of the cases to which it is especially adapted. After the lateral is brought into place, the spur is fastened either into the lateral itself or a cuspid, either or both.

*Dr. Wilson.*—In such a case, you would put two spurs in?

*Dr. Ainsworth.*—I would use one or two, as the case seemed to require.

*Dr. Mead.*—In cases where the teeth have been drawn back very far, after getting them into position, I usually strike up a metal cap, about one-third the length of the teeth, and that holds them firm and fast.

*Dr. Andrews.*—I can speak well of the spur. I have used it for twelve or fifteen years. Dr. Cutler calls a case to my mind where I used it seven or eight years ago. My method was to drill a cavity in the tooth slightly smaller than the screw I was to use. I then dipped its end in sandarac varnish, and screwed it into place. You would be surprised to see some of these screws that were put in long ago; they have been allowed to remain, and no decay has taken place around them. I think the spurs were put in to hold back laterals which had been prominent—just such a case as Dr. Brackett speaks of. I drilled into the lateral, the gold screw was screwed into the hole, and the end rested against the cuspid. These spurs that Dr. Baker has shown seem to be in both teeth; I use them only in one tooth, and I always place them so that they are not in the way of articulation. I may have been indebted to Dr. Baker for the idea, for we have often exchanged views on such subjects.

However, I should be loath to drill into a sound tooth for the purpose of putting in a spur. It might not do any harm, but if we don't do it, we are certainly not running any risk.

To my mind, for a retaining apparatus to be used on the anterior teeth, there is nothing so valuable as a band, with little prongs on either side. I recently saw a case which was under the care of a New York dentist, and there were six of these prongs, holding the regulated teeth in position, and holding them well.

*Dr. Ainsworth.*—There are a great many cases where the patient objects to anything that is conspicuous—anything that shows at all in the front of the mouth—and I think in such cases the spurs are a very great advantage. They can be worn for years, entirely concealed, and without any discomfort to the patient. I think in two-thirds of the cases where we want to use one we will find a cavity already existing. I should never hesitate to drill a cavity on the inside of a tooth, where it is out of sight, and can be perfectly cared for after we get through with the spur.

I make a point, when regulating, of carrying a tooth a little farther than I wish to have it remain, trusting that when the spur is removed, the tooth will naturally drop back a little.

I would say further, in regard to this regulating band and the ligatures, it is possible to produce a very great deal of suffering in tying up teeth or wedging teeth, and it is necessary to use a great deal of caution. There are a great many different ways in which a tooth can be tied up, and I venture to say that there are very few cases which cannot be tied in a positive way so that the ligature will not slide up under the gum, and produce unnecessary pain.

William H. Potter, D.M.D.,
*Editor American Academy of Dental Science.*

